# Does the Low-Carbon City Pilot Policy Improve the Urban Land Green Use Efficiency?—Investigation Based on Multi-Period Difference-in-Differences Model

**DOI:** 10.3390/ijerph20032704

**Published:** 2023-02-02

**Authors:** Shuchen Niu, Xiang Luo, Tiantian Yang, Guodong Lin, Chongming Li

**Affiliations:** College of Public Administration, Central China Normal University, Wuhan 430079, China

**Keywords:** low-carbon pilot policy, ULGUE, SFA, multi-period difference-in-differences model, SDM-DID

## Abstract

Improving urban land green use efficiency (ULGUE) is an effective way to increase social, economic, and ecological benefits and achieve regional sustainable development goals. This study takes three batches of low-carbon pilot cities construction as a quasi-natural experiment and investigates the impact of low-carbon pilot construction on ULGUE through the multi-period difference-in-differences method and spatial Dubin difference model (SDM-DID). The results show that (1) from 2006 to 2019, ULGUE in China increased. From the aspect of space, ULGUE in China gradually decreased from west to east, showing an obviously high agglomeration phenomenon in Beijing–Tianjin–Hebei and the Pearl River Delta; (2) after the robustness test, parallel trend test, and endogenous test, it is found that the conclusion that the low-carbon pilot construction can effectively improve ULGUE is still relevant and can indirectly improve ULGUE in the local region through fund allocation, talent gathering, and industrialization; and (3) the national ULGUE has significant positive spatial correlation. The results of the SDM-DID model confirm that the low-carbon pilot policy can produce the significant spatial spillover and drive the common advance of ULGUE in neighboring regions. Therefore, the resources and environmental conditions in each city are supposed to be taken into full consideration theoretically. Furthermore, it is necessary to effectively promote the development of ULGUE by strengthening the linkage of green production factors between different cities, so as to make meaningful contributions to promoting China’s overall green development.

## 1. Introduction

Land is an important material carrier for urban economic, ecological, and social development. The rational and efficient use of land is a fundamental way to alleviate resource difficulties, ensure economic prosperity, and achieve the sustainable development of land space. However, with the accelerating process of urbanization, the construction land scale has expanded rapidly. From 2000 to 2020, the urban construction land area in China increased by 163.89% (the data are from *China City Statistical Yearbook*). The land area to be built in the development zone reached 67,000 hectares in 2020, and the idle land increased by nearly 40% compared with 2021 (http://www.gov.cn/xinwen/2021-01/13/content_5579414.htm (accessed on 20 September 2021)). The rapid expansion of cities has caused increasingly serious problems, such as inefficient land use and the disorderly development of marginal areas [1,2,3,4]. At the same time, extensive land use also damages the ecological balance of cities. As the main source of new urban land, agricultural land can also produce a lot of emissions in the transformation process, aggravating urban environmental problems [5,6,7]. There is little doubt that the problem of low efficiency and pollution caused by the disorderly expansion of cities has become the biggest obstacle to the realization of high-quality development in China.

In order to improve resource utilization efficiency and advocate a sustainable development mode, the Chinese government has introduced a series of policies and measures to improve the urban green development level, of which the low-carbon pilot policy has attracted much attention. In September 2007, China clearly advocated the development of a low-carbon economy at the 15th Asia–Pacific Economic Cooperation (APEC) leaders’ meeting. With the meeting as an opportunity, the National Development and Reform Commission issued the Notice on the National Low-Carbon City Pilot Work and the Notice on the Pilot Work of Low-Carbon Provinces and Cities in 2010, the Notice on the Second Batch of Low-Carbon Provinces and Cities in 2012, and the Notice on the Third Batch of National Low-Carbon Cities Pilot Work and 2017, and successively identified 80 cities, such as Shanghai, Tianjin, and Shenzhen, as national low-carbon pilot cities, leading the country in exploring a new model of low-carbon development. Since then, active exploration has been carried out all over the country. Jiangsu, Guangdong, Hubei The information is from Implementation Opinions of Jiangsu Government on Accelerating the Establishment and Improvement of a Green and Low-Carbon Recycling Development Economic System, the 14th Five-Year Plan on Ecological Civilization Construction in Guangdong, and the Implementation Plan for Green Buildings Creation in Hubei, respectively. Other provinces (prefecture-level cities) successively introduced action plans for low-carbon construction in terms of intensive land use, green buildings, and other aspects in order to comprehensively implement the green low-carbon development model.

With the deepening of the green development concept, the relationship between low-carbon policy and urban land green use is becoming closer [8,9,10]. The low-carbon policy can not only reduce the carbon emissions of land use by optimizing the urban spatial structure [11,12] but also restrain urban expansion by increasing areas of urban green belts, thereby helping relieve the impact of climate change [13], which has a more significant effect in larger cities [14]. However, most of the existing studies focus on the measurement and spatio-temporal analysis of urban land green use efficiency itself from the perspective of low-carbon, and research on the influence mechanism and spatial spillover effect is relatively rare. Therefore, this study analyzes the impact of low-carbon pilot policy on urban land green use efficiency (ULGUE) in 266 Chinese cities by studying the following three issues: (1) Can the low-carbon pilot policy improve ULGUE? (2) If so, how is it implemented? (3) Is there a significant spatial spillover effect? By answering these questions, the current situation of green land use in China can be comprehensively evaluated, which is beneficial to establishing and improving the existing policy evaluation system. In addition, it can speed up the improvements regarding performance evaluations of low-carbon pilot policies and provide theoretical support for the expansion of low-carbon pilot policies. Finally, it will help to clarify the key urban development mechanism and accelerate the realization of sustainable development goals.

Building upon the concept of the Kuznets curve proposed by economist Simon Smith Kuznets in 1955, the relationship between environmental quality and per capita income was named the Environmental Kuznets Curve (EKC) by Panayotou in 1993. The prediction results of the above hypothesis had been confirmed by actual conditions in western countries; therefore, many scholars have tried to test its applicability in China in recent years. Due to China’s increasing attention to its own environmental problems, administrative units at different levels have been changing from an interactive game to cooperation, which has advanced the arrival of an “inflection point” for the inverted “U” curve. This not only helps China build strong confidence in dealing with environmental and development issues but also shows its exemplary role in global environmental protection and green transformation and development, which is also of great significance to other developing countries. Obviously, whether from the perspective of practice or research value, the problems derived from interregional development in China are no longer limited to the country itself, they are increasingly becoming major concerns for the whole world. The starting point of this study is very consistent with the above background. The study of the spatial spillover effect of low-carbon pilot policy provides strong support for understanding the importance of public policy in green development.

The rest is arranged as follows: Section 2 reviews the relevant literature, which lays the vital foundations for the following mechanism analysis; Section 3 theoretically analyzes the effect mechanism for ULGUE; Section 4 provides the research design, research method, data resources, variable selection, etc.; Section 5 gives our empirical analysis, including basic robustness and endogeneity tests; Section 6 explores the impact mechanism and spatial spillover effect of low-carbon pilot policy on ULGUE; and Section 7 shows our conclusions and relevant policy recommendations.

## 2. Literature Review

Fully understanding the concept, measurement methods, and driving factors of urban land green use efficiency is of great significance for testing the relationship between the low-carbon pilot policy and ULGUE. In terms of concept, ULGUE is the ratio of input factors (land and other factors) of the land use system and land use output (including economic and ecological values) under certain production technology conditions. This reveals that ULGUE emphasizes the coupling of economy and ecological environment, and this study takes into comprehensive consideration economic and environmental factors. Unlike the traditional land use efficiency (ULUE), which only takes economic benefits as “desirable” output, urban land green use efficiency (ULGUE) takes urban pollution index as “undesirable” output when ensuring the rationalization of input and output, and establishes a land use evaluation system combining efficiency and green factors [15]. In terms of measurement methods, urban land use efficiency (ULUE) mostly uses multi-input methods to measure land use efficiency, such as data envelopment, entropy, and stochastic frontier analyses [16,17,18,19], relieving the difficulty of conducting a multi-dimensional evaluation. With the continuous development of a green concept and measurement model, social and environmental costs should also be considered on the basis of considering economic benefits. With the help of the Slacks-based measure (SBM) model, which measures super efficiency based on unexpected output and stochastic frontier analysis, pollution indicators, such as industrial “three wastes” and carbon emissions, are set as unexpected output to achieve a more comprehensive ULGUE measurement [15,19]. Therefore, the expression of ecological significance is strengthened while the land economic effect is focused on.

The driving factors are roughly divided into three categories: (1) Regional policy. The regional policy covers high-tech zone policies and special economic zone policies [19,20]. The local government integrates resources according to regional characteristics and strengthens the input–output ratio of unit land area. However, the regional policy sometimes has negative effects. “Land finance” has been proved to have a direct impact from improvement to inhibition on ULGUE, producing a spatial spillover effect, and the long-term effect is more obvious than the short-term effect [21]. (2) Industry cluster. In the industry cluster process, two results of a cluster economy and cluster diseconomy emerge. In the process of an industry cluster, there will be two results, namely an agglomeration economy and agglomeration diseconomy, which will have positive and negative impacts on ULGUE. The cluster economy can be understood by Jacobs’ externality theory [22], namely that the integrated development of different industries produces the knowledge spillover effect and innovation effect, which not only improve the productivity of enterprises but also promote the economic benefits of land. The cluster diseconomy means that based on the premise of scarcity regarding land resources, an excessive industry cluster will lead to problems, such as a rise in the land use costs of enterprises and a decline in ecological environment quality, resulting in a simultaneous reduction in land economic benefits and environmental benefits [23]. (3) Social development. The existing studies have shown that population migration, urbanization, and regional economic integration are closely related to ULGUE [24,25,26,27]. In addition, urban infrastructure construction will also have a significant impact on ULGUE [28,29]. For example, the research of Xiao et al. [29] shows that urban roads can effectively attract and accelerate factors flow, optimize the land use model, and improve ULGUE.

## 3. Effect Mechanism Analysis

As a basic human value, environmental protection has been recognized by the whole of society; therefore, it has become one of the basic political goals of government. The implementation of a low-carbon pilot policy can not only directly affect ULGUE through “emission reduction” and “greening”, it also indirectly affects ULGUE through technological and structural changes. In order to fully reveal the influence mechanism of low-carbon pilot projects on ULGUE, the direct effect and indirect effect will be analyzed below.

The low-carbon pilot policy directly affects ULGUE through “emission reduction” and “greening”. In terms of “emission reduction”, in low-carbon pilot areas, the government usually introduces strict environmental regulations to ensure that the wastes in the production procedures of enterprises in the region meet emission requirements. At this time, manufacturers tend to directly allocate parts of funds as pollution control expenditure, which encourages enterprises to make technological progress in pollution control and emission reduction, reduce pollution emissions, and increase ULGUE accordingly [30]. In terms of “greening”, under the triple pressure of low-carbon targets and the assessment and public demand of the National Development and Reform Commission (NDRC), local governments in pilot areas tend to increase their investment in basic facilities, such as “urban green lungs”, while “reducing emissions”. Therefore, urban ecological resources will continue to be enriched, and the local environment will be improved with the acceleration of the decomposition and absorption of pollutants, promoting ULGUE progress [31].

**H1.**  *The low-carbon pilot policy can significantly improve ULGUE*.

The low-carbon pilot policy indirectly affects ULGUE through technological and structural effects. Through the technological effect, the low-carbon pilot policy can effectively improve the urban innovation level. Technological innovation is a key channel to improve resource allocation efficiency and labor production in the whole of society, meaning it is an important driving force for high-quality development [32,33,34]. Combining the existing literature and economic logic, we found that the low-carbon pilot policy mainly promoted technological innovation through talent gathering and capital allocation effects. Regarding fund allocation, during the construction of low-carbon pilot cities, the local government at all levels adjusts the financial expenditure structure according to their own conditions, increases R&D subsidies for enterprises, and reduces taxes on innovative enterprises. While mitigating the costs and risks of innovation subjects, they convey positive information to society and promote regional scientific and technological innovation. Talent gathering is considerable because, as a direct factor input, talents are conducive to accelerating local independent innovation and catalyzing the green innovation process of enterprises. At the same time, as carriers of new knowledge and skills, innovators can effectively promote enterprises’ learning abilities, thereby transforming, integrating, and applying new technologies, while providing intellectual support for emissions reduction and efficiency increases. Technical innovation can effectively improve ULGUE [35,36]. First of all, scientific and technological innovation can effectively promote productivity increases. By improving economic development, the pilot cities enhanced their comprehensive strength, attracted more high-quality investment projects, and increased the intensity of input–output per unit area of land, improving ULGUE in low-carbon pilot cities. More importantly, high-end and new technologies can promote the rapid development of communication, energy, transportation, etc., constantly enhancing the urban accessibility and thereby increasing attractiveness for external investment. Therefore, increases in regional incomes lead to changes in land use structures, elevating ULGUE.

**Figure 1:**
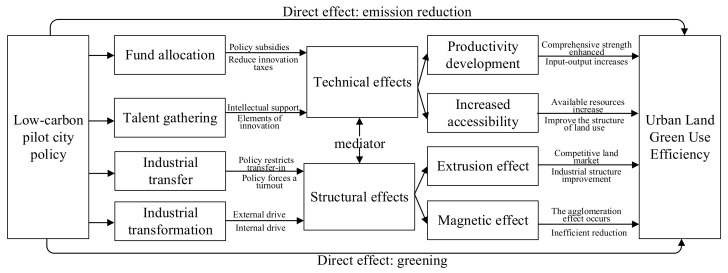
Analysis of impact mechanism of low-carbon pilot policy on ULGUE.

Through the structural effect, a low-carbon pilot policy can significantly optimize the urban industrial structure. The optimization of industrial structures is essential to improving resource allocation levels among industries and promoting the optimal ratio of input factors to output factors. The low-carbon pilot policy mainly optimizes the industrial structure through industrial transfer and industrial transformation. Regarding industrial transfer, according to the “Pollution Paradise Hypothesis”, the low-carbon pilot policy will lead to the transfer of high-pollution enterprises from pilot areas to non-pilot areas because more relaxed environmental policies in non-pilot areas can accommodate these enterprises [37]. At the same time, due to policy restrictions, it is difficult for low-carbon pilot cities to undertake high-pollution enterprises transferred from other regions in order to optimize regional industrial structures. Next, according to existing studies, industrial transformation can be divided into externally driven and internally driven. Regarding externally driven transformation, according to the “Porter hypothesis” [38], when regional economic development is high, the environmental standard of production can be improved accordingly, with only enterprises whose innovation cost is less than innovation income surviving, resulting in a regional industrial transfer. Regarding internally driven transformation, when the demand structure changes, a hasty industrial transfer will result in huge sunk costs and goodwill losses. Some enterprises will choose to make corresponding adjustments to their industrial structure and technology investment to cope with environmental changes, thus completing the process of regional industrial transformation. The optimization of the industrial structure can promote ULGUE. On the one hand, the non-renewable nature of land itself will lead to the competitive use of resources in the land market, increasing the price of land and enterprise production costs. As a result, the “crowding-out effect” of industrial restructuring will force enterprises with high-pollution and low-income levels to move away from the area. The economic and environmental benefits of the area and ULGUE will be improved [29]. On the other hand, thanks to the development of information technology, production factors, development policies, and other information in various regions can be circulated at a high speed. Low-carbon pilot cities have a “magnetic attraction effect”, attracting industries with high input–output returns to continue to concentrate, resulting in a cluster effect. The proportion of land used by inefficient industries is reduced, promoting ULGUE. The effect mechanism, including direct and indirect effects, is shown in Figure 1.

**H2.**  *Low-carbon pilot policies indirectly improve ULGUE through technological and structural effects*.

However, the low-carbon pilot policy may also have a negative effect, leading to ULGUE reduction. On the one hand, harsh environmental protection policies have a negative impact on regional economic development [39,40]. For example, in their research, Martin et al. [39] found that the implementation of differentiated “emission reduction” policies in the EU increased energy prices in the region, leading to higher production costs and thereby affecting the regional ULGUE. On the other hand, a perfect design and good intentions may not generate good results. Similarly, enterprises may also accelerate resource consumption and increase urban pollution emissions due to a fear that green economic policies could damage their future earnings [41], resulting in ULGUE reduction.

The main reason for the divergence in the above studies lies in the differentiated approaches to achieving emission reduction goals. In foreign countries, in the view of emission reduction policies increasing the production costs of energy-intensive industries in the region, emission reduction regions will transfer carbon-intensive industries to neighboring regions in order to achieve the coexistence of emission reduction goals and profits maximization. This process will not only increase global greenhouse gas emissions but also affect economic benefits in emission reduction regions [39]. This phenomenon is called carbon leakage, whereby emission reduction areas take measures that lead to carbon emission increases in other non-emission-reduction areas. The research has revealed that the European Union and other developed countries have produced a very serious “carbon leakage” phenomenon [42], increasing carbon emissions in surrounding countries that are used to produce cheap export products, thereby implicating themselves in the development dilemma [43,44], resulting in difficulties for ULGUE improvement.

Different from other countries, China has incorporated environmental performance assessments into the hard indicators of local government performance assessments (http://www.gov.cn/jrzg/2013-12/09/content_2545183.htm (accessed on 20 September 2021); https://www.spp.gov.cn/dj/c100027/201711/t20171109_320723.shtml (accessed on 22 September 2021); http://hzedz.hanzhong.gov.cn/jjkfqzf/gwhwj/201901/t20190119_565959.shtml (accessed on 22 September 2021)), which means that regional officials need to take into account environmental quality on the basis of regional economic development, which leads to the phenomenon that surrounding regions do not fully accept the carbon-intensive industries transferred from pilot areas. Other regions will force pilot cities to implement technology upgrades and industrial transformation while ensuring their own economic development and environmental benefits. While relieving the “carbon leakage” phenomenon in low-carbon pilot cities [45], the policy promotes the common progress of ULGUE in China through the spillover effects of technological upgrading and industrial transformation [46,47,48,49,50]. According to the International Energy Agency (IEA) report (https://www.iea.org/reports/global-energy-review-co2-emissions-in-2021-2 (accessed on 22 September 2021)), the overall carbon emission intensity in China decreased by 40% compared with 2000, while its intensity in developed countries has only decreased by 3% since 2010, which also supports the above view of this study.

**H3.**  *The low-carbon pilot policy can produce the positive spillover effect to improve ULGUE in nearby areas*.

## 4. Research Design

### 4.1. Stochastic Frontier Model

For the measurement of input–output efficiency, the current methods mainly include stochastic frontier analysis (SFA) and data envelopment analysis (DEA). However, DEA cannot take the random disturbance term into account, while SFA can test its own parameters and applicability, which can reduce the influence caused by uncontrollable factors by effectively distinguishing management error terms and statistical error terms. Therefore, this paper constructs the input–output SFA for ULGUE based on the Cobb–Douglas production function. The model is as follows:

(1) $$Y_{it}\;=F\left(L_{it}\;,\;t;\;\beta \right)\exp\left(V_{it}-K_{it}\right)$$

where i is an city; t is time; $y_{it}$ is the comprehensive output per area; $L_{it}$ is the input factor vector, β is the coefficient vector to be estimated; $V_{it}$ is a random error term of the independent identical distribution; and $K_{it}$ is the technical ineffectiveness degree. When $K_{it}$ is 0, it refers to the overall full effective state; when $K_{it}$ increases, it means that the land use efficiency level is decreasing in areas farther away from the frontier. This paper uses gross non-agricultural product to reflect the expected output in land use, and also uses industrial wastes to represent the unexpected output. The logarithmic form of the model is as follows:

(2) $$\ln\left(y_{it}\right)\;=\beta_{0}+\beta_{1t}\ln\left(G_{it}\right)+\beta_{2t}\ln\left(H_{it}\right)+\beta_{3t}\ln\left(F_{it}\right)+V_{it}-K_{it}$$

where $y_{it}$ represents the comprehensive output per area of city i in t period; $\beta_{0}$ is the intercept item; $G_{it}$, $H_{it}$, and $F_{it}$ refer to the fixed-asset investment per area (100 million CNY/km^2^), unit employees at the end of year per area (people/km^2^), and scientific expenditure per unit area (100 million CNY/km^2^), respectively; $\beta_{1}$, $\beta_{2}$, and $\beta_{3}$ refer to the output elasticity of capital, labor, and science, respectively; and $V_{it}$ and $K_{it}$ are the random error term and invalid rate term, respectively.

Urban land green use efficiency (ULGUE) represents the ratio of the expected value of actual output for urban land use to the expected output when $K_{it}$ = 0.

(3) $${\mathrm{ULGUE}}_{it}\;=\;\exp\left(-K_{it}\right)\;=\;\frac{E(Y_{it}|K_{it},\;L_{it})}{E(Y_{it}|K_{it\;}=0,\;L_{it})}$$

where ULGUE is urban land green use efficiency when $K_{it}\;$≥ 0, 0 < ${\mathrm{ULGUE}}_{it}\;$≤ 1. Meanwhile, when the generalized likelihood ratio is used for testing the applicability of SFA, the LR statistic is as follows:

(4) $$\mathrm{LR}=-2\left[L\left(w_{0}\right)-L\left(w_{1}\right)\right]$$

where LR~$\chi_{1-\alpha }^{2}$(K) means that the above formula meets the mixed $\chi^{2}$ distribution; *K* is the freedom degree and *α* is the significant level; $L\left(w_{0}\right)$ is the constrained logarithm likelihood function value; and $L\left(w_{1}\right)$ is the unconstrained logarithm likelihood function value.

According to the above and the existing literature [10,15,19], this study constructs an indicator system to measure ULGUE (Table 1). Since SFA is a multiple-input and single-output model, a comprehensive index of expected output is constructed by the entropy weight method for ULGUE measurement.

### 4.2. Multi-Period DID Model

In the view of previous studies, the difference-in-differences model has become the mainstream method for policy effect evaluation [12,16]. The practice of using the difference-in-differences model to evaluate policy effect can provide better suggestions for the government to formulate policies. This study takes the low-carbon pilot policy in China as the cutting-in point to explore the policy effect on China’s urban land use efficiency. Since low-carbon pilot cities are set up in different batches at different times, this study adopts the multi-period difference-in-differences method to set the following model:

(5) $${\mathrm{ULGUE}}_{it}=\beta_{0}+\beta_{1}did_{it}+\lambda X_{it}+v_{i}+\mu_{t}+\varepsilon_{it}$$

In the above formula, i refers to city, *t* refers to year, and the dependent variable ${\mathrm{ULGUE}}_{it}$ is the urban land green utilization efficiency of city i in year i. $did_{it}$ is a difference-in-differences estimation value, and the variables are set as follows: the pilot city treat is set as 1, and the non-pilot city is set as 0. If city i is set as a pilot city in year t, this year and subsequent years are each assigned to 1, otherwise they are 0. time×treat=did is applied to three batches of low-carbon pilot cities in 2010, 2012, and 2017. The result that the interaction coefficient is significantly positive indicates that the establishment of pilot cities can accelerate the development of ULGUE. The control variable is $\lambda X_{it}$. In addition, the time t and city i are both fixed, represented by $v_{i}\;$and $\mu_{t}$, respectively.

### 4.3. Spatial Econometric Model

With the method of Jia et al. [51] for reference, we add the spatial lag term of the explained variable on the basis of the spatial dual difference model (SLX-DID) studied by Diao et al. [52], and we obtain the spatial Dubin difference model (SDM-DID) to test the spatial spillover effect of pilot cities.

(6) $${\mathrm{ULGUE}}_{it}=\alpha_{0}+\rho \sum_{j}W_{ij}{\mathrm{ULGUE}}_{it}+\alpha_{1}did_{it}+\theta \sum_{j}W_{ij}did_{it}+\lambda X_{it}+\delta \sum_{j}W_{ij}X_{it}+v_{i}+\mu_{t}+\varepsilon_{it}$$

where ${\mathrm{ULGUE}}_{it}$ represents the urban land green use efficiency; $did_{it}$ is the core independent variable, representing the interactive item of policy implementation in pilot cities; $X_{it}$ is the control variable; and W represents the spatial weight matrix. The fixed region, fixed time, and residual error are represented by $v_{i}$, $\mu_{t}$, and $\varepsilon_{it}$, respectively.

### 4.4. Variable Selection and Data Demonstration

Dependent variable: ULGUE based on the stochastic frontier model.

Core independent variable: did. The variable is the interactive item.

Control variables: Based on the existing literature, the following control variables are selected: (1) Financial health (*fd*). Good financial health is conducive to driving local support for environmental protection. Therefore, in the process of promoting the low-carbon pilot policy, the impact of government capacity cannot be ignored. This paper uses the value of fiscal expenditure minus fiscal revenue to reflect financial health [5] (2) Opening up (*fdi*). The introduction of foreign capital is conducive to the development of urban economies and industry; however, it may also worsen local pollution due to the introduction of high-carbon industries. Therefore, opening up is measured by the actual amount of foreign capital used in the year [19]. (3) Urban housing investment (*Inres*). In recent years, China’s real estate market has been hot, and investment has been increasing, which has squeezed investment in science and technology and the environment. Therefore, this paper explores the impact of urban housing investment on ULGUE through the real estate development investment (*Inres*). In order to verify the impact of urban ecological environment and information development on ULGUE, the following control variables are added for research. (4) Ecological environment (*ee*). This is characterized by urban green land per capita. (5) Urban informatization (*informar*). This is represented by the total amount of urban telecommunication services.

Mediators: (1) The structural effect (*ind*). The upgrading of an industrial structure refers to the transformation process of the urban industrial form from low-end to high-end. This study adopts the ratio of the output value of tertiary industry to that of secondary industry to express the industrial upgrading degree, reflecting the industrial structure adjustment. *z*1, *z*2, and *z*3 represent the ratio of output values in the primary industry, secondary industry, and tertiary industry to GDP, respectively. (2) The technological effect. The capital allocation and talent gathering aspects of technological innovation are respectively reflected by the proportion of scientific expenditure in finance (*tec*) and the proportion of employees involved in information transmission, computer services, and the software industry in unit employees at the end of year (*itcss*). The descriptive statistical results of all variables are shown in Table 2.

**Figure 2:**
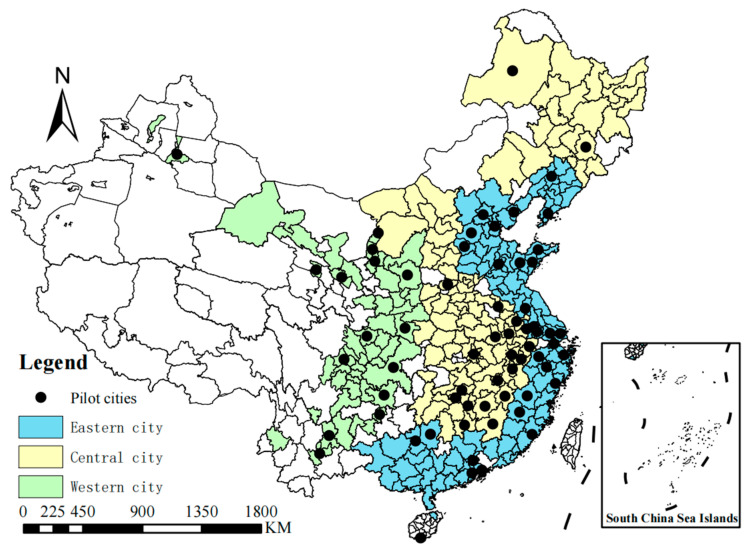
Distribution of low-carbon pilot cities.

Because some data before 2006 had different statistical calibers, and because China suffered from the COVID-19 pandemic after 2019, the economic data are biased, which can easily cause estimation errors. Generally, the difference-in-differences model is used to evaluate the effects of random experiments or natural experiments (such as the adjustment of laws and regulations), and a period of time is needed to observe such effects. Data at several years before and after the experiment are required to changes of dependent variables before and after the experiment. Therefore, this study selects cities at prefecture level and above in China from 2006 to 2019 for the research. After excluding cities that were withdrawn during the study period or have considerable amounts of missing data, we finally retained 266 cities as research units (excluding Tibet, Hong Kong, Macao, and Taiwan). The data are mainly from the China Urban Statistical Yearbook, China Urban Construction Statistical Yearbook, and the statistical yearbooks of provinces and cities. Because strong panel data are required for spatial measurement, a few missing parts are completed by interpolation. In order to avoid the influence of heteroscedasticity during the analysis, the numerical variables are treated by logarithm. The distribution for three batches of low-carbon pilot cities in China is shown in Figure 2.

## 5. Empirical Analysis

### 5.1. ULGUE Measurement Results

Based on the panel data on input–output for 266 cities in China from 2006 to 2019, the random frontier analysis is conducted through frontier 4.1 software. The measured ULGUE results are shown in the following Table 3.

**Figure 3:**
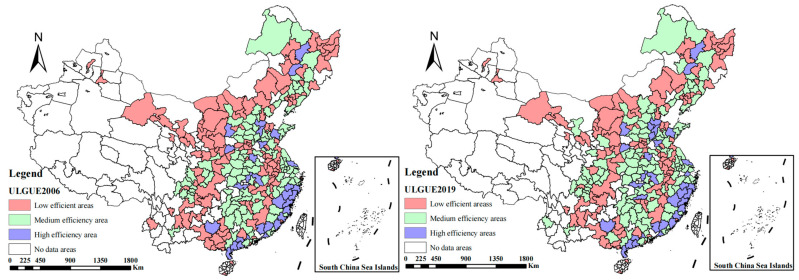
ULGUE distribution changes in 266 prefecture-level cities in 2006 and 2019 in China.

From the test results, we see that the LR test of the one-sided error was successful, and the gamma value is 0.758, indicating that technical inefficiency accounts for a lot in the error term. The parameter coefficients all passed the *t* test, and the parameter estimation is accurate at a 99% confidence level. Therefore, it is scientific to use SFA to calculate ULGUE in this paper. From the elasticity of input–output, β1 = 0.057, β2 = 0.085, and β3 = 0.014, indicating that the comprehensive output increases by 0.057, 0.085, and 0.014 respectively, when the fixed-asset investment per unit area, unit employees at the end of year per area, and scientific expenditure per unit area all increase by 1. Then, according to the natural breakpoint method, ULGUE results are divided into three grades: high level, medium level, and low level. The results are shown in the following Figure 3.

**Figure 4:**
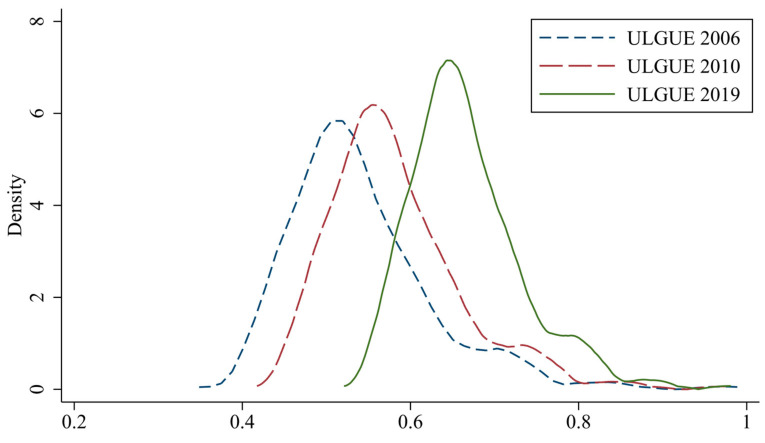
Dynamic temporal evolution characteristics of ULGUE.

In terms of space, ULGUE in China increased in 2019 compared with 2006; ULGUE showed obvious clusters in Beijing–Tianjin–Hebei, the Pearl River Delta, and other regions, with the overall trend decreasing from east to west. Compared with central and western regions, the eastern region had significant advantages in technological innovation, industrial upgrading, and environmental awareness due to trade, shipping, policy, and other factors, whereas ULGUE in the eastern region has been in a dominant position in China for a long time. In terms of time, the gravity center of the overall curve in Figure 4 shifts to the right from 2006 to 2019, with greater changes from 2010 to 2019, which shows that overall ULGUE in China has been rising during the study period; the right tailing in 2019 is larger and shows significantly thicker and higher results in 2019 compared with 2006 and 2010, which means there are more high-efficiency regions. The above changes show that the low-carbon development concept has begun to play an increased role since 2010, with the national production efficiency and ecological level elevated at a higher speed.

### 5.2. Multi-Period DID Regression

#### 5.2.1. Basic Regression

Stata 16 is used to complete the basic DID regression. The model in the 1st column of Table 4 presents the basic regression result based on time–control and individual–control. Then, control variables are added in turn to the model from the 2nd to 6th columns based on basic regression. The results show that the coefficients of core independent variables are significantly positive, indicating that the low-carbon pilot policy has a significant role in promoting ULGUE, which is consistent with hypothesis 1. At the same time, it is worth noting that *fdi* has a stable and significant positive impact on ULGUE. For China, during periods of rapid economic growth, foreign capital introduction is conducive to the development of urban economic industries and promotes the growth of ULGUE. *fd* has a stable and significant negative impact on ULGUE, indicating that with the continuous increase in fiscal deficit and pressure on government expenditure, ULGUE growth is constrained. *inres* has a stable and significantly positive impact on ULGUE, indicating that the increase in investment in real estate development may squeeze the investment in science, technology, and the environment, leading to a decrease in ULGUE.

#### 5.2.2. Parallel Trend Test

**Figure 5:**
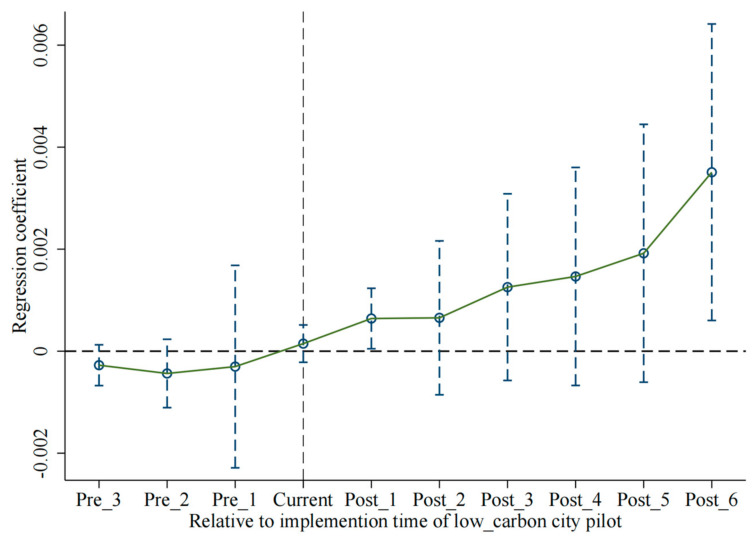
The dynamic effect tests.

We use the event analysis method to test the parallel trend. Specifically, it is to compare the experimental group (after the pilot launch) and control group (before the pilot launch). If there are significant differences before and after the pilot launch, it indicates that the policy implementation has an impact on pilot cities. As shown in Figure 5, Pre_3~Pre _1 represent the three years before policy implementation, when regression coefficients are not significant, though with a fluctuating trend. Current is the policy implementation year and Post_1~Post_6 are the six years after policy implementation with significantly positive coefficients, which show significant differences in these three years. This shows cities in the experimental group and cities in the control group have changed significantly after the policy implementation, meeting the parallel trend assumption.

#### 5.2.3. Robustness Test

The above basic regression results and parallel trend test results show that the construction of low-carbon pilot cities can effectively improve ULGUE. However, in order to exclude the interference of other confounding factors on conclusions, the robustness test is to be conducted below for ensuring the stability of the results. In order to avoid the interference of individual outliers on results, this study mainly conducts the sample data screening for the robustness test, and conducts the regression after 1%, 5%, and 10% tail-shrinking treatment for dependent variables and control variables, respectively. As shown in Table 5, the columns 1, 3, and 5 are regression results without control variables, whereas columns 2, 4, and 6 are regression results with control variables. All these results have passed the significance test at the 1% level, indicating that the low-carbon pilot city policy helps to improve ULGUE, which supports the above conclusions.

#### 5.2.4. Endogenous Testing

The endogeneity in this study mainly originates from missed variables and reciprocal causation. In terms of reciprocal causation, low-carbon pilot cities tend to have high ULGUE; however, cities with higher ULGUE are not always selected as low-carbon pilot cities. This study counts ULGUE in 2010, 2012, and 2017, years when low-carbon pilot cities were established. The results show that among the top 10 cities ranked by ULGUE, only Jinhua as a pilot city ranks in the top 10 in three batches of 2010, 2012, and 2017. It is revealed that there is no inevitable link between ULGUE and becoming a low-carbon pilot city. At the same time, in order to avoid endogenous problems caused by missed variables, this study added a series of control variables in the regression. Furthermore, the difference-in-differences method is selected due to the external policy shock, which is conducive to avoiding endogeneity in the econometric model.

In the view of the application system for being a low-carbon pilot city, the propensity score-matching–difference-in-differences (PSM-DID) regression model is adopted to further strengthen the randomness of sample selection in the control group and the experimental group, so as to alleviate the endogeneity. The common methods include Markov distance matching, nearest neighbor matching, caliper (radius) matching, kernel matching, and so on. In this study, Markov distance matching and kernel matching are selected for data screening. After the screening data results are obtained, the multi-period DID is used for regression. The results are shown in Table 6, which proves that the basic regression results are robust. However, it should be noted that this method can only alleviate the endogeneity caused by observable variables, and it is difficult to deal with the endogeneity caused by unobservable variables.

## 6. Further Analyses

### 6.1. Influence Mechanism Research

In the theoretical analysis part, we concluded that the impact of low-carbon pilot cities on ULGUE is mainly achieved through the structural effect and technological effect. In order to verify these two impact channels, we selected related variables on technological innovation and industrial structure for the empirical test.

#### 6.1.1. Industrial Structure

For testing the actual effect of an industrial structure as an impact mechanism, the first step should be to test whether industrial structure has a significant impact on ULGUE. The possibility that the industrial structure change has a significant impact on ULGUE is conducive to further testing whether the implementation of low-carbon city pilot policies has an impact on urban industrial structure. If the above estimated results can pass the significance test, it is believed that the indirect effect of low-carbon pilot city construction on ULGUE changes exists by affecting the industrial structure.

First, the GDP proportion of three industries and industrial upgrading indicators are added to the basic regression successively, as shown in columns 1, 2, 3, and 4 of Table 7. The GDP proportion in the primary industry, the GDP proportion in the tertiary industry, and industrial upgrading have a positive impact on ULGUE; the GDP proportion in the secondary industry has a significantly negative impact on ULGUE. The above results show that the industrial structure change has a significant impact on ULGUE, having passed the significance test. At present, China’s secondary industry is dominated by high-carbon, high-pollution middle- and low-end manufacturing industries, so the increase in GDP proportion in the secondary industry will have a negative impact on ULGUE.

Next, we need to test whether the implementation of a low-carbon city pilot policy has an impact on urban industrial structure. We replaced core independent variables with the proportion of three industries and industrial upgrading, and used formula (1) to conduct the regression. The results are shown in columns 5, 6, 7, and 8 in Table 7. The construction of low-carbon cities will indeed significantly reduce the GDP proportion in the secondary industry; moreover, the GDP proportion in the primary industry, the GDP proportion in the tertiary industry, and the industrial upgrading actually have the effect of promotion, and the industrial upgrading passed the significance test.

To sum up, the implementation of a low-carbon pilot policy has indeed had an impact on urban industrial structure, and industrial upgrading plays a significant role in promoting ULGUE. Therefore, we can conclude that the effect low-carbon pilot city construction has an indirect impact on ULGUE changes by affecting the industrial structure.

#### 6.1.2. Technological Innovation

To test the indirect effect of technological innovation, according to the above analysis, we are supposed to first test whether technological innovation has a significant impact on ULGUE, and then test whether the implementation of a low-carbon city pilot policy has an impact on urban technological innovation. The results are shown in Table 8. Both talent gathering and capital allocation significantly improved ULGUE and passed the 1% significance test (columns 1 and 2 in Table 8), and the construction of low-carbon pilot cities can indeed improve urban talent gathering and capital allocation levels (columns 3 and 4 of Table 8), reflecting the positive impact of the pilot policy on technological innovation.

To sum up, the implementation of low-carbon pilot policy has a significant impact on scientific and technological innovation levels in cities, and the scientific and technological innovation level has been proved to drive ULGUE to develop upward. Therefore, we can conclude that there is an indirect effect regarding the construction of low-carbon pilot cities and ULGUE changes through affecting scientific and technological innovation levels.

### 6.2. Research on Spatial Spillover Effect

According to the mechanism analysis, we can see that the current official promotion contest system in China can effectively alleviate the “carbon leakage” problem in low-carbon pilot cities. The mechanism research in this paper confirmed that the low-carbon pilot policy can significantly improve the industrial structure and innovation levels for low-carbon pilot cities, while the structural effect and technological effect have obvious spillover effects [48,49,50], so as to boast the development of surrounding cities and improve the ULGUE of adjacent areas.

First of all, after testing the global Moran’s I from 2006 to 2019, the results indicated that ULGUE has a spatial correlation, which can be further verified. The results are actually shown in the following Table 9: the significant spatial autocorrelation is shown in the adjacency matrix and inverse distance matrix, with the trend rising year by year. Secondly, through LR and Hausman tests, the assumption that SDM-DID will degenerate into SEM or SLM has been rejected. Therefore, the spatial Dubin model with double fixed effects is mainly analyzed below.

According to the first law of geography, all things in the world are connected universally and objectively. The establishment of the spatial weight matrix is the basis of spatial measurement. Common weight matrices include the adjacency matrix, economic distance matrix, geographical distance matrix, and economy–geography matrix. Each of the above matrices has its own applicable scenario for spatial measurement; therefore, this study chooses the most commonly used adjacency matrix for subsequent research according to its situation.

As shown in the first column of Table 10, the coefficient of low-carbon pilot policy is 0.0063, which is statistically significant at the 1% level, and this coefficient and significance level are remarkably higher than that of the basic regression. The regression coefficients 0.00947 and 0.04899 in columns 3 and 4, respectively, also pass the significance test, which reveals that both the regions have a positive impact on ULGUE. The coefficient *Wx* of *did* is significantly positive, indicating that low-carbon pilot cities have a positive spatial spillover effect on ULGUE, and that the low-carbon pilot policy can significantly improve ULGUE in adjacent areas. During the construction of low-carbon pilot cities, the pilot cities may have played a “leading role” in promoting the flow of high-quality elements among cities, as well as surrounding cities’ direct or indirect learning from pilot cities through the structural effect and technological effect. Therefore, their ULGUE can be enhanced. What is interesting is that in the SDM-DID model, *informar*, has a negative impact on ULGUE in other regions while having a significantly positive impact on ULGUE in this region. This may be due to the “siphon effect” caused by the improvements in urban informatization level, which constantly absorbs the development resources of surrounding regions, deteriorating ULGUE in other regions while improving its own ULGUE. That *ee* is always positive indicates the green area is indeed conducive to improving China’s overall environmental situation and urban green development.

## 7. Discussion

This study selected 266 cities at prefecture level and above as samples and adopted muti-period DID and SDM-DID models to investigate the impact of low-carbon pilot construction. The results show that the low-carbon pilot policy is able to improve the ULGUE of pilot cities through technical and structural effects and has a positive impact on the ULGUE of surrounding cities.

(1) Channel mechanism. First of all, the low-carbon pilot policy aims at drawing up low-carbon development planning, formulating supporting policies, and establishing an industrial system characterized by low-carbon emissions. Based on urban land green use, it is intended to speed up the optimization of urban industrial structure, so as to continuously reduce urban environmental pollution and resource consumption while increasing urban economic development scale, which refers to the dialectical unity of environment and development. More importantly, technological innovation, as a key channel to improve the productivity of the whole society, is an important driving force for green development. With the deepening of the implementation of low-carbon pilot policy, the capital and talent levels have been continuously improved, the cost and benefits beneath low-carbon production have been consistently rationalized, and the productivity and green development have been mutually promoted and improved.

(2) Spatial spillover. Firstly, due to its unique national condition, China has seen barriers to the “industrial transfer” between cities, namely that low-carbon pilot cities cannot transfer high-pollution enterprises to non-pilot cities arbitrarily, and that non-pilot cities tend to prudently accept carbon-intensive industries due to official promotion and policy limitations. Then, the unique “reverse driving” mechanism is formed to urge pilot and non-pilot cities to jointly improve urban green development levels, which the significantly positive results of *did*s in Table 10 have proved. This is helpful for China to embark on a civilized development path of production development, ensuring a rich life and sound ecology in the future. Secondly, the research exerts a vital impact on global climate change. Since the Kyoto Protocol in 1997, major developed countries have transferred a large number of carbon-intensive industries to developing countries in order to meet their own emission reduction standards. The phenomenon of “carbon leakage” has failed to make an obvious effect on the remission of global climate change. The low-carbon pilot policy in China has been proved to generate a significant effect on China’s overall green development. If this experience is applied to neighboring countries and even the world, more positive effects are to be seen on global climate change. However, the path to realize the combination of its operation mechanism and national conditions of each country, so as to exert more positive impacts on global climate change, is a notable direction for future research.

## 8. Conclusions and Suggestions

Based on the panel data of 266 prefecture-level cities from 2006 to 2019, this study used the low-carbon pilot policy in China as a quasi-natural experiment and established the multi-period difference-in-differences and SDM-DID models to verify that the construction of low-carbon pilot cities has a significant impact on ULGUE.

The specific conclusions are as follows: (1) In general, the construction of low-carbon pilot cities has a significantly positive impact on ULGUE. This conclusion is still valid after a series of robustness tests that objectively prove the feasibility of low-carbon-pilot city construction for promoting urban ecological, economic, and social benefits. (2) The impact of low-carbon pilot policies on ULGUE has both direct and indirect effects. The low-carbon pilot construction has been proved to directly improve ULGUE, and also indirectly improve ULGUE through technological and structural effects, indicating that the impact of low-carbon pilot city construction on ULGUE is multi-dimensional and multi-channel. (3) The construction of low-carbon pilot cities can not only improve local ULGUE, but also result in a significantly positive impact on nearby cities. Based on these conclusions, this study proposes the following suggestions:

(1) The low-carbon pilot policy diffusion is implemented steadily and orderly. The core of the pilot policy is to follow the gradual reform model, which is conducive to avoiding the uncontrollable risks caused by large-scale transformation while fostering the advance of green and efficient development models. The above conclusions have proved that the low-carbon pilot policy not only contributes to local ULGUE improvement but also has a significant spatial spillover effect to improve the ULGUE of neighboring cities. This shows that the low-carbon pilot policy has played a significant positive role in ensuring a city’s “green and efficiency” levels. Therefore, the pilot cities can summarize and condense their successful experiences, continuously expand the pilot scope of low-carbon cities, and gradually promote the policy to the whole country. Meanwhile, we should correctly grasp the positive spatial spillover effect generated by the construction of low-carbon pilot cities, promote cross-regional cooperation between cities, and strengthen the flow of green production factors among different cities to promote steady improvements in ULGUE and make important contributions to the realization of the “dual carbon” goal and global climate change objectives;
(2) The channel mechanism is used to provide path support for the further optimization of low-carbon pilot policies. From the perspective of effectively improving technological innovation and industrial structure, the low-carbon pilot policy will play a more far-reaching and lasting role in ULGUE through optimizing the role of low-carbon cities. On the one hand, the innovation support is supposed to be provided by increasing innovation investment, talent support, and infrastructure construction. Furthermore, the creation of an innovative atmosphere and improvements in urban innovation ability are to be achieved by building a knowledge-sharing platform and promoting the free flow of factor resources. On the other hand, it is necessary to reasonably plan industrial development goals and realize the effective transformation of secondary industry to tertiary industry, high-additional-valued industry to low-additional-valued industry, and high-pollution industry to low-carbon environmentally protective industry in the region, as well as improving large-scale industrial operations with the aid of provincial development parks and national high-tech industrial parks and other carriers to promote the transformation of regional industrial structure. Global climate change originates from the industrial revolution, including its effects on industrial structure changes and resource consumption. The further optimization of operation channels though a low-carbon pilot policy is certainly conducive to mitigating global climate change.

There are still several limitations in this study. First of all, due to the difficulty in obtaining data, such as greenhouse gas emissions and soil erosion, the unexpected output in ULGUE is only measured by using industrial wastes. There is still room for improvement in this study, regardless of whether it is infinitely close to the actual level. Therefore, future research should focus more on continuously improving the composite output index, so as to interpret ULGUE from a more comprehensive perspective. Secondly, the spatial effect verified by spatial Durbin difference model is jointly reflected by pilot cities and non-pilot cities, which leads to difficulties in stripping and quantifying the net effects of the pilot policy on non-pilot cities. In subsequent research, on the basis of continuously improving the ULGUE indicator system, we will pay more attention to the net effect of low-carbon pilot policy on non-pilot cities through more appropriate methods in order to help policy makers formulate relevant strategies and achieve sustainable urban development goals.

## Figures and Tables

**Table 1:** Evaluation index system of ULGUE.

Variables	First-Level Indicator	Secondary-Level Indicator	Unit
Input	Capital	Fixed-asset investment per area	100 million CNY/km^2^
	Labor	Unit employees at the end of year per area	10,000 people/km^2^
	Science and technology	Scientific expenditure per area	100 million CNY/km^2^
Output	expected output	Non-agricultural GDP per area	100 million CNY/km^2^
	Unexpected output	Industrial sulfur dioxide emission per area	Ton/km^2^
		Industrial wastewater emission per area	Ton/km^2^
		Industrial fumes emission per area	Ton/km^2^

**Table 2:** Descriptive Statistics.

Symbol	Sample Size	Mean	Standard Deviation	Min.	Max.
ULGUE	3724	0.605	0.0860	0.370	0.981
*fdi*	3724	9.833	1.900	0	14.94
*informar*	3724	12.37	1.011	8.686	16.45
*fd*	3724	0.521	0.227	−0.541	0.945
*inres*	3724	13.85	1.359	9.268	17.61
*ee*	3724	2.254	1.029	−0.966	6.197
*z*1	3724	12.77	8.143	0.0300	49.89
*z*2	3724	39.20	9.754	8.580	83.52
*z*3	3724	48.03	10.60	10.68	90.97
*ind*	3724	0.907	0.493	0.0943	5.168
*tec*	3724	0.196	0.0444	0.0199	0.387
*itcss*	3724	0.0126	0.00921	0.000459	0.109

**Table 3:** Estimation results of stochastic frontier equation.

Symbol	Coefficient	Standard-Error	T-Ratio
β0	−0.984	0.026	−37.202
β1	0.057	0.008	7.023
β2	0.085	0.005	18.745
β3	0.014	0.003	4.516
sigma-squared	0.037	0.002	19.259
gamma	0.758	0.009	85.041
mu	0.336	0.022	15.307
eta	0.033	0.002	19.354
Log-likelihood function	2813.202		
LR test of the one-sided error	2135.056		

**Table 4:** Basic regression results.

Variables	(1)	(2)	(3)	(4)	(5)	(6)
	ULGUE	ULGUE	ULGUE	ULGUE	ULGUE	ULGUE
*did*	0.00113 **	0.00099 **	0.00109 **	0.00114 **	0.00105 **	0.00108 **
	(2.38)	(2.08)	(2.29)	(2.40)	(2.26)	(2.32)
*fdi*		0.00062 ***	0.00057 ***	0.00050 ***	0.00074 ***	0.00074 ***
		(5.07)	(4.70)	(4.03)	(6.09)	(6.09)
*informar*			0.00088 ***	0.00084 ***	0.00107 ***	0.00105 ***
			(3.49)	(3.33)	(4.35)	(4.24)
*fd*				−0.00631 ***	−0.00931 ***	−0.00937 ***
				(−4.01)	(−5.98)	(−6.02)
*inres*					−0.00352 ***	−0.00356 ***
					(−12.94)	(−13.09)
*ee*						0.00093 **
						(2.49)
Constant	0.54049 ***	0.53499 ***	0.52492 ***	0.52919 ***	0.56971 ***	0.56897 ***
	(1488.47)	(467.65)	(169.13)	(161.56)	(127.30)	(126.95)
Observations	3724	3724	3724	3724	3724	3724
R-squared	0.979	0.979	0.979	0.980	0.980	0.980
Number of city	266	266	266	266	266	266
City	Yes	Yes	Yes	Yes	Yes	Yes
Year	Yes	Yes	Yes	Yes	Yes	Yes

Notes: ***, ** are significant at the levels of 1%, 5%, respectively. All of the following tables also apply.

**Table 5:** Robustness test.

Variables	(1)	(2)	(3)	(4)	(5)	(6)
	ULGUE	ULGUE	ULGUE	ULGUE	ULGUE	ULGUE
*did*	0.00120 **	0.00107 **	0.00283 ***	0.00251 ***	0.00277 ***	0.00230 **
	(2.30)	(2.10)	(3.65)	(3.29)	(2.68)	(2.24)
Constant	0.54120 ***	0.57135 ***	0.54550 ***	0.57808 ***	0.55161 ***	0.57230 ***
	(1358.77)	(113.03)	(920.11)	(71.86)	(699.77)	(50.99)
Observations	3724	3724	3724	3724	3724	3724
R-squared	0.975	0.976	0.939	0.942	0.877	0.880
Number of city	266	266	266	266	266	266
Control	No	Yes	No	Yes	No	Yes
City	Yes	Yes	Yes	Yes	Yes	Yes
Year	Yes	Yes	Yes	Yes	Yes	Yes

Notes: ***, ** are significant at the levels of 1%, 5%, respectively. All of the following tables also apply.

**Table 6:** PSM-DID regression results.

Variables	(1)	(2)	(3)	(4)
	Mahalanobis Distance		Kernel Matching	
	ULGUE	ULGUE	ULGUE	ULGUE
*did*	0.00139 ***	0.00142 ***	0.00139 ***	0.00142 ***
	(2.83)	(2.96)	(2.82)	(2.97)
Constant	0.54004 ***	0.57238 ***	0.53998 ***	0.57215 ***
	(1457.72)	(126.28)	(1460.97)	(126.28)
Observations	3626	3626	3630	3630
R-squared	0.979	0.981	0.979	0.981
Number of city	266	266	266	266
Control	Yes	Yes	Yes	Yes
City	Yes	Yes	Yes	Yes
Year	Yes	Yes	Yes	Yes

Notes: *** are significant at the levels of 1%. All of the following tables also apply.

**Table 7:** Empirical analysis of impact of industrial structure on ULGUE.

Variables	(1)	(2)	(3)	(4)	(5)	(6)	(7)	(8)
	ULGUE	ULGUE	ULGUE	ULGUE	*z*1	*z*2	*z*3	*ind*
*did*	0.00103 **	0.00107 **	0.00104 **	0.00103 **	0.153	−0.291	0.136	0.0336 **
	(2.232)	(2.306)	(2.257)	(2.235)	(0.959)	(−0.903)	(0.527)	(2.142)
*z*1	0.000311 ***							
	(6.329)							
*z*2		−0.000116 ***						
		(−4.730)						
*z*3			6.01 × 10^−5^ **					
			(1.975)					
*ind*				0.00122 **				
				(2.417)				
Constant	0.558 ***	0.566 ***	0.571 ***	0.567 ***	35.35 ***	48.04 ***	16.63 ***	2.028 ***
	(116.7)	(120.1)	(127.3)	(123.3)	(22.84)	(19.15)	(5.341)	(13.35)
Observations	3724	3724	3724	3724	3724	3724	3724	3724
R-squared	0.981	0.981	0.981	0.981	0.428	0.685	0.491	0.526
Number of city	266	266	266	266	266	266	266	266
Control	Yes	Yes	Yes	Yes	Yes	Yes	Yes	Yes
City	Yes	Yes	Yes	Yes	Yes	Yes	Yes	Yes
Year	Yes	Yes	Yes	Yes	Yes	Yes	Yes	Yes

Notes: ***, ** are significant at the levels of 1%, 5%, respectively. All of the following tables also apply.

**Table 8:** Empirical analysis of impact of technological innovation on ULGUE.

Variables	(1)	(2)	(3)	(4)
	ULGUE	ULGUE	*itcss*	*tec*
*did*	0.00095 **	0.00079 *	0.00364 ***	0.01350 ***
	(2.03)	(1.69)	(7.99)	(7.02)
*itcss*	0.03467 **			
	(2.00)			
*tec*		0.02139 ***		
		(5.23)		
Constant	0.56809 ***	0.56385 ***	0.02530 ***	0.23921 ***
	(126.21)	(123.37)	(5.75)	(12.84)
Observations	3724	3724	3724	3724
R-squared	0.981	0.981	0.096	0.215
Number of city	266	266	266	266
City	Yes	Yes	Yes	Yes
Year	Yes	Yes	Yes	Yes
Control	Yes	Yes	Yes	Yes

Notes: ***, **, and * are significant at the levels of 1%, 5%, and 10%, respectively. All of the following tables also apply.

**Table 9:** Global Moran’s I from 2006 to 2019.

	Adjacency Matrix			Inverse Distance Matrix		
	I	z	*p*	I	z	*p*
2006	0.342	8.521	0.000	0.069	10.155	0.000
2007	0.342	8.532	0.000	0.070	10.175	0.000
2008	0.343	8.543	0.000	0.070	10.193	0.000
2009	0.343	8.553	0.000	0.070	10.211	0.000
2010	0.344	8.562	0.000	0.070	10.228	0.000
2011	0.344	8.571	0.000	0.070	10.244	0.000
2012	0.344	8.58	0.000	0.070	10.260	0.000
2013	0.345	8.588	0.000	0.070	10.275	0.000
2014	0.345	8.596	0.000	0.071	10.289	0.000
2015	0.345	8.603	0.000	0.071	10.302	0.000
2016	0.346	8.61	0.000	0.071	10.315	0.000
2017	0.346	8.616	0.000	0.071	10.328	0.000
2018	0.346	8.622	0.000	0.071	10.34	0.000
2019	0.347	8.628	0.000	0.071	10.351	0.000

**Table 10:** Verification of low-carbon pilot city construction and ULGUE empirical results.

Variables	(1)	(2)	(3)	(4)	(5)
	Main	Wx	LR_Direct	LR_Indirect	LR_Total
*did*	0.00630 ***	0.00632 ***	0.00947 ***	0.04899 ***	0.05846 ***
	(8.79)	(4.77)	(10.38)	(8.33)	(9.02)
*fdi*	−0.00026	−0.00067 *	−0.00050 **	−0.00368 ***	−0.00418 ***
	(−1.27)	(−1.96)	(−2.23)	(−2.65)	(−2.76)
*informar*	0.00263 ***	−0.00014	0.00322 ***	0.00858 ***	0.01181 ***
	(6.76)	(−0.18)	(7.00)	(2.64)	(3.33)
*fd*	0.01559 ***	0.00232	0.01963 ***	0.06348 ***	0.08311 ***
	(6.00)	(0.59)	(7.15)	(4.37)	(5.26)
*ee*	0.00782 ***	0.00365 ***	0.01057 ***	0.04293 ***	0.05351 ***
	(13.67)	(3.47)	(15.73)	(9.82)	(11.15)
*inres*	0.00733 ***	−0.00257 ***	0.00823 ***	0.01376 ***	0.02198 ***
	(16.35)	(−4.24)	(17.77)	(6.93)	(10.18)
rho	0.79179 ***				
	(80.87)				
sigma2_e	0.00008 ***				
	(40.26)				
Constant	0.01156				
	(1.21)				
Observations	3724	3724	3724	3724	3724
R-squared	0.076	0.076	0.076	0.076	0.076
Number of city	266	266	266	266	266

Notes: ***, **, and * are significant at the levels of 1%, 5%, and 10%, respectively. All of the following tables also apply.

## Data Availability

The data presented in this study are available on request from the corresponding author.
